# Draft Genome Sequence of *Xanthomonas arboricola* pv. juglandis J303, Isolated from Infected Walnut Trees in Southern Chile

**DOI:** 10.1128/genomeA.01085-17

**Published:** 2017-09-28

**Authors:** Julio Retamales, Cristopher Segovia, Romina Alvarado, Pablo Nuñez, Javier Santander

**Affiliations:** a Agroadvance Laboratory, Peñaflor, Chile; b Instituto de Ciencias Naturales, Universidad de Las Americas, Viña del Mar, Chile; c Faculty of Sciences, Universidad Mayor, Huechuraba, Chile; d Marine Microbial Pathogenesis and Vaccinology Laboratory, Department of Ocean Sciences, Memorial University of Newfoundland, St. John's, Canada

## Abstract

Here, we report the draft genome sequence of *Xanthomonas arboricola* pv. juglandis J303, isolated from infected walnut trees in southern Chile. The size of the genome is 5,066,424 bp with a G+C content of 65.4%. *X. arboricola* pv. juglandis J303 has several genes related to virulence, antibiotic resistance, and copper resistance.

## GENOME ANNOUNCEMENT

The Gram-negative bacterium *Xanthomonas arboricola* pv. juglandis is the etiological agent of walnut blight disease. Walnut blight is the main disease affecting walnut production (1), and losses during a wet spring can exceed 80% if it is not controlled (2, 3). In Chile, *X. arboricola* pv. juglandis outbreaks affect southern areas of the country. The current control of *X. arboricola* pv. juglandis is done through widespread utilization of copper-based agrochemicals (4). The efficiency of copper-based agrochemicals has decreased due to the emergence of resistant *X. arboricola* pv. juglandis strains (5–8). Also, utilization of heavy metals like copper in food products could have agronomic and sanitary consequences, as it lacks acceptance by consumers. Here, we describe the draft genome of *X. arboricola* pv. juglandis J303, isolated from infected walnut trees in southern Chile and utilized as a host for the propagation of bacteriophages (9).

*X. arboricola* pv. juglandis J303 was routinely grown in yeast peptone glucose broth (YPG) (10 g peptone, 5 g yeast extract, 5 g glucose per liter) with aeration (180 rpm) at 28°C. The genomic DNA was extracted according to Wilson (10) and purified using silica (11). Sequencing was performed using the Illumina MiSeq next-generation sequencing platform (Universidad Mayor, Center for Genomics and Bioinformatics, Huechuraba, Chile) and paired-end libraries. Low-quality sequences were examined by FastQC version 0.10.1 (12). The sequences were trimmed and assembled using the CLC Genomics Workbench version 10.1.1 (Qiagen) *de novo* tool, resulting in 161 contigs over 1 kb, with an *N*_50_ value of 66,602 bp. The total length of the draft genome of *X. arboricola* pv. juglandis J303 was 5,066,424 bp with a G+C content of 65.4%.

The assembled sequences were annotated by NCBI Prokaryotic Genome Annotation Pipeline (https://www.ncbi.nlm.nih.gov/genome/annotation_prok). The tRNAs were detected by tRNA scan-SE version 1.3 (13) and the rRNAs with RNAmmer (14). A total of 4,329 coding sequences, 234 pseudogenes, 1 complete rRNA operon (5S-16S-23S), 49 tRNAs, and 38 noncoding RNAs were predicted by the pipeline.

We identified several genes related to copper and antibiotic resistance. For instance, the copper resistance protein B (CrpB) (NCBI reference sequence number WP_053054037), the cytoplasmic copper homeostasis protein (CutC) (WP_016904876), and the magnesium and cobalt efflux protein (CorC) (WP_016902067) were present in *X. arboricola* pv. juglandis J303, suggesting that this strain, like others previously isolated in other locations, has acquired copper resistance. This correlates with the inefficacy of copper-based agrochemicals to treat walnut blight disease (4) and indicates that the use of biological control treatments, like the utilization of bacteriophages, could be a sustainable option for controlling *X. arboricola* infection in trees.

### Accession number(s).

This whole-genome shotgun project (BioProject number PRJNA310300) has been deposited at DDBJ/EMBL/GenBank under accession number LSGZ00000000. The version described here is the first version, LSGZ01000000.
